# Recovery from Liver Failure and Fibrosis in a Rat Portacaval Anastomosis Model after Neurointermediate Pituitary Lobectomy

**DOI:** 10.1155/2021/5529784

**Published:** 2021-12-09

**Authors:** Martín Muñoz-Ortega, Noé Macías-Segura, Javier Ventura-Juárez, Manuel Enrique Ávila-Blanco, Leonardo D. Ponce-Damian, Daniel González-Blas, Esperanza Sánchez-Alemán, Andrés Quintanar-Stephano

**Affiliations:** ^1^Departamento de Química, Centro de Ciencias Básicas, Universidad Autónoma de Aguascalientes, Aguascalientes, PC 20100, Mexico; ^2^Departmento Fisiología y Farmacología, Centro de Ciencias Básicas, Universidad Autónoma de Aguascalientes, Aguascalientes, PC 20100, Mexico; ^3^Departamento de Morfología, Centro de Ciencias Básicas, Universidad Autónoma de Aguascalientes, Aguascalientes, PC 20100, Mexico; ^4^Departamento de Patología, Hospital General ISSSTE, Aguascalientes, PC 20010, Mexico

## Abstract

Liver diseases, including cirrhosis, viral hepatitis, and hepatocellular carcinoma, account for approximately two million annual deaths worldwide. They place a huge burden on the global healthcare systems, compelling researchers to find effective treatment for liver fibrosis-cirrhosis. Portacaval anastomosis (PCA) is a model of liver damage and fibrosis. Arginine vasopressin (AVP) has been implicated as a proinflammatory-profibrotic hormone. In rats, neurointermediate pituitary lobectomy (NIL) induces a permanent drop (80%) in AVP serum levels. We hypothesized that AVP deficiency (NIL-induced) may decrease liver damage and fibrosis in a rat PCA model. Male Wistar rats were divided into intact control (IC), NIL, PCA, and PCA+NIL groups. Liver function tests, liver gene relative expressions (IL-1, IL-10, TGF-*β*, COLL-I, MMP-9, and MMP-13), and histopathological assessments were performed. In comparison with those in the IC and PCA groups, bilirubin, protein serum, and liver glycogen levels were restored in the PCA+NIL group. NIL in the PCA animals also decreased the gene expression levels of IL-1 and COLL-I, while increasing those of IL-10, TGF-*β*, and MMP-13. Histopathology of this group also showed significantly decreased signs of liver damage with lower extent of collagen deposition and fibrosis. Low AVP serum levels were not enough to fully activate the AVP receptors resulting in the decreased activation of cell signaling pathways associated with proinflammatory-profibrotic responses, while activating cell molecular signaling pathways associated with an anti-inflammatory-fibrotic state. Thus, partial reversion of liver damage and fibrosis was observed. The study supports the crucial role of AVP in the inflammatory-fibrotic processes and maintenance of immune competence. The success of the AVP deficiency strategy suggests that blocking AVP receptors may be therapeutically useful to treat inflammatory-fibrotic liver diseases.

## 1. Introduction

Liver diseases (e.g., cirrhosis, viral hepatitis, and hepatocellular carcinoma) create an enormous burden on the global healthcare system, accounting for approximately two million deaths per year. In Mexico, it is the third leading cause of death in men and seventh in women [1]. Currently, research into new treatments for liver diseases is of great importance. Liver disease and fibrosis are the result of chronic inflammatory processes, which are independent of its etiology (e.g., drugs, alcohol history, hepatitis virus B and C, obesity, and autoimmunity). Liver inflammation and fibrosis are initiated in response to permanent liver damage with excessive accumulation of collagen overdegradation [2–5]. During the initial stage, liver damage induces the release of proinflammatory cytokines like IL-1, TNF-*α*, and IL-8 [2, 3, 6]. If the liver damage persists, the inflammatory response includes infiltration of lymphocytes, plasma cells, polymorphonuclear cells, histiocytes, fibroblast, and development of regeneration nodules, vascular distortion, and fibrosis [3]. During persistent inflammation, the hepatic stellate cells (HSCs) are activated by different kind of factors: cytokines (IL-1, TNF-*α*, and IL-8), growth factors (TGF-*β*, PDGF, and ET-1), and factors from the endothelial cells, Kupffer's cells, hepatocytes, and platelets. All these factors induce differentiation of HSCs into myofibroblasts with properties such as proliferation, contraction, fibrogenesis, and expression of type I collagen (*Col1al*) and alpha-smooth muscle actin (*α*-SMA) [2, 4], thus making HSCs one of the main responsible factors for liver fibrosis and liver failure. Currently, liver fibrosis treatment is done to revert fibrosis and to restore the liver functions [2, 7–10].

There are several models of experimental fibrosis-cirrhosis, which have physio-pathogenic characteristics of the human cirrhosis. Some models of the experimental liver fibrosis are induced by (1) CCl_4_ administration for eight weeks, (2) acetaminophen administration for ten weeks, (3) ethanol administration for more than ten weeks, (4) hypercaloric diets for more than 12 weeks [5, 7, 8, 11], and (5) portacaval anastomosis (PCA) [12–14].

In rats, PCA induces a decrease in serum albumin, hyperbilirubinemia, and increased serum levels of bile acids, alkaline phosphatase (AP), aspartate aminotransferase (AST), alanine aminotransferase (ALT), lactate dehydrogenase (LDH), creatinine, urea, and ammonium [13–15] with clear signs of liver histopathological damage such as hepatocytic necrosis and apoptosis, portal inflammation, biliary proliferation, steatosis, and fibrosis. All these observations indicate that the PCA model reproduces the clinical and histopathological signs of chronic liver disease [13–15].

In recent years, neuro-immune-endocrine interactions in inflammatory diseases have allowed for a better understanding of pathological regulatory processes. In this context, arginine vasopressin (AVP), also called antidiuretic hormone, synthesizes in the paraventricular and supraoptic nuclei of the hypothalamus, passes through the axons to the posterior lobe of the pituitary gland (neurohypophysis), and releases into the blood. Corticotropin-releasing hormone (CRH) and AVP of the hypothalamic paraventricular parvocellular neurons play an important role in coordinating hypothalamic-pituitary-adrenal axis activity during stress, inflammation, and autoimmune diseases [16, 17]. AVP also has several other activities that include diuresis inhibition, contraction of vascular smooth muscle cells, and liver glycogenolysis. AVP is also involved in several brain functions that affect memory, anxiety, and depression [18, 19]. Presently, most of the evidence indicates that AVP acting directly on different cells of the immune system is a proinflammatory hormone [20–23]. We have shown that animals that underwent surgical removal of the neurohypophysis (NIL) showed a permanent decrease in AVP and oxytocin (OXT) blood levels (80% and 90% below the normal ranges, respectively) [24]. Experiments from others [25] and from our lab have demonstrated that decreased AVP serum levels (NIL-induced) diminish humoral and cellular immune responses [20, 21, 26, 27]. Profibrogenic properties of AVP on the heart, liver, and kidney have been demonstrated [28–32]. In addition, we have reported that AVP deficiency promotes the reduction of collagen deposits in a CCl_4_ cirrhosis hamster model and restores the balance between metalloproteinases and tissue inhibitors of metalloproteinases (TIMPs) [33]. All this evidence supports that AVP is a major player in the regulation of immune responses and fibrosis; however, less is known on cell and molecular mechanisms through AVP deficiency may modulate the immune responses. Thus, in this work, we study the effects of AVP deficiency (NIL-induced) on liver inflammation, tissue damage, and fibrosis in the PCA rat model.

## 2. Materials and Methods

### 2.1. Animals

Male Wistar rats (*Rattus norvegicus*) at 6-8 weeks old (200-250 g body weight) from our Animal Care Facility were used. Animals were treated according to the Institutional Normative Welfare Standards of the Autonomous University of Aguascalientes and the official Mexican regulations (NOM-062-ZOO-1999). Experimental protocols were approved by the Institutional Bioethics Committee.

**Figure 1:**
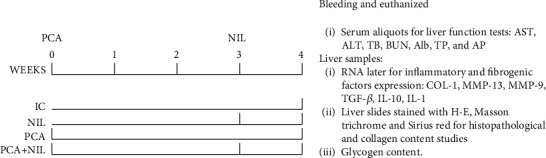
Experimental schedule. Rats were divided into intact control (IC), neurointermediate pituitary lobectomy (NIL), portacaval anastomosis (PCA), and the PCA+NIL groups. PCA and NIL surgeries were performed at weeks 0 and 3, respectively. At week 4, all animals were anesthetized, bled, and euthanized. Serum aliquots were used for assessing functional liver test, tissue liver samples were used to assess inflammatory and fibrogenic factors expression, histopathological studies and collagen and glycogen contents. *n* = 4 − 6 animals/group.

Animals were maintained in a 12 h/12 h light/dark cycle and 21-22°C room temperature and fed with Purina Rat Chow (Ralston Purina Company, St. Louis, MO, USA). Food and water were provided *ad libitum*. The rats were divided into the following four groups (4-6 animals/group): (1) intact control (IC), (2) NIL, (3) PCA, and (4) PCA+NIL. In the PCA+NIL group, NIL surgery was performed three weeks after PCA.  Figure 1 shows the experimental schedule.

### 2.2. Portacaval Anastomosis (PCA)

PCA was performed at week 0 in the PCA and PCA+NIL groups (Figure 1). The PCA microsurgery technique used was an adaptation of those described by Aller et al. [13] and Padilla-Sánchez [34]. Rats were anesthetized with a mixture of ketamine (80%) and xylazine (20%) (Cheminova, Mexico) (1 *μ*L/g of body weight/i.p.), and a laparotomy was performed to access the abdominal organs. Under stereomicroscope (Zeiss OPMI-19 FC at 6x magnification), the portal vein was dissected, followed by the right kidney vein and the inferior cava vein. The cava vein was dissected from above the left kidney vein to where the cava vein is covered by the liver lobule. The right kidney vein and the ends of the isolated cava vein were transiently occluded by a gentle pull of removable threads. On the left side of the dissected cava, a window was opened and washed with heparin-saline solution (1%) (Inhepar, Heparina, Pisa, Mexico). The dissected portal vein was temporarily occluded with a surgical clip at its union with the splenic vein, and the vein was tied and cut below the knot at the hilum liver level. The remnant vein blood was washed with heparin-saline solution. The open end of the portal vein was then anastomosed with the cava vein window. The PCA was performed in less than 15 min. After surgery, the animals were placed in a recovery box with clinical oxygen and controlled temperature. For infection prevention, animals were injected with penprocillin (6000 IU, i.m.) (Pisa, Mexico) once a day for three days. For analgesia, sodium metamizole (Pharmalife, Mexico) (10 mg/kg i.m.) was administrated once daily for three days. After anesthesia recovery, the animals were put in cages with food and water *ad libitum*.

### 2.3. Neurointermediate Pituitary Lobectomy (NIL)

NIL surgeries were performed on the NIL and PCA+NIL groups at week 3 of the experiment (see experimental schedule in Figure 1).

The method employed has been described in our previous work [35]. Fifteen minutes prior to anesthesia, 0.06 mg atropine/s.c. (Atropisa, Pisa, Mexico) was injected to prevent excessive airway secretion. Animals were anesthetized with a mixture of ketamine 80% and xylazine 20% (1 *μ*L/g body weight/i.p.). Removal of the neurointermediate pituitary lobe (neural and intermediate lobes) was performed under a dissecting microscope (Zeiss OPMI-19 FC at 6x magnification) through the parapharyngeal-transoccipital-sphenoidal approach. After direct viewing of the neurointermediate lobe, it was gently aspirated using a bent needle as follows. The neck was shaved, and the animal was placed into dorsal decubitus on the operating table. With the upper incisor fork, the head was fixed to the table, while the legs were fastened with threads to the lateral edges of the table. The trachea was cannulated through the snout. The surgical approach to the pituitary gland included the following steps: (1) asepsis and cutting of the skin on the anterior aspect of the neck, (2) identification of the left digastric muscle, (3) blunt separation of the digastric muscle central tendon, (4) placement of retractors to get a wider view of the bottom of the opening, (5) identification of the distal end of the pterygoid process and the long neck muscles, (6) identification and cleaning of the basioccipital and basisphenoid bones, (7) display of the occipital-sphenoid joint, (8) trepanation of the skull in the center of the occipital-sphenoid joint until the pituitary capsule can be viewed, (9) cutting of the pituitary capsule at its most posterior end, (10) elevation of the adenohypophyseal lobe and visual identification of the intermediate and neural lobes of the hypophysis, and (11) gentle aspiration of the neurointermediate lobe with a bent needle

The total time of the surgery was within 15 min, and animals were fully recovered within 40 min. For infection prevention, animals were injected with penprocillin after the surgery (6000 IU, i.m./3 days). For analgesia, sodium metamizole (10 mg/kg/i.m./2 days) was used.

All groups were euthanized at the fourth week of experimentation. Before euthanasia, animals were anesthetized with sodium pentobarbital (Maver, China), bled from the abdominal aorta, and the serum was aliquoted and frozen at −70°C until the liver function tests. Samples of liver tissue were immediately immersed in RNA later (Invitrogen, Thermo Fisher Scientific, USA) and processed to determine pro- and anti-inflammatory and pro- and antifibrogenic gene expression (relative levels). For histopathological study, liver tissue samples were fixed in 10% neutral formalin solution in paraffin, cut into 5 *μ*m thick slices, mounted on slides, and stained with hematoxylin-eosin (HE) for histopathological study and Masson's trichrome and Sirius Red stains for fibrosis area and collagen content estimation [36]. Fuji software was used to determine the percentage of fibrosis area [37] from Sirius Red-stained slides observed under polarized light at 400x magnification. The histopathological study was performed under a Nikon light microscope Optiphot-2.

### 2.4. Liver Function Tests

Serum samples were defrosted, and the following markers of hepatic function were assessed: AST, ALT, AP, LDH, total bilirubin, total protein, albumin, and urea serum levels. All tests were performed using kits from Spinreact (Girona, Spain) following the manufacturer's instructions. Samples were read in a spectrophotometric semiautomatic bts-350 analyzer (Biosystems, Quezon City, Philippines).

### 2.5. RNA Isolation and Determination of Gene Expression by Real-Time qPCR

Total RNA was isolated from 100 mg of liver samples with the Jena Bioscience Isolation System (Jena Bioscience, Jena, Germany), following the manufacturer's protocol. Total RNA was quantified with a NanoDrop 2000 (Thermo Scientific, Waltham, MA, USA). Reverse transcription was performed with 1 *μ*g of total RNA using the GoScript Reverse Transcription System (Promega) for real-time quantitative PCR, which was analyzed using qPCR GreenMaster with UNG-clear (Jena Bioscience, Jena, Germany) in a StepOne machine (Applied Biosystems) under the following conditions: 50°C for 2 min, 95°C for 3 min, 40 cycles of 95°C for 45 sec, and 60°C for 45 sec. Oligonucleotides were designed to target type I collagen (COL-I), MMP-13, MMP-9, TGF*β*, IL-10, IL-1, and *β*-actin (as a reference control) (Table 1). Relative expression level was normalized with *β*-actin as reference gene, and differences were determined using the 2^−ΔΔCt^ method.

### 2.6. Statistical Analyses

All data were evaluated for Gaussian distribution using the Kolmogorov-Smirnov normality test. Multiple comparisons between the groups were performed for each parameter. One-way ANOVA and Tukey's post hoc test for parametric data or the Kruskal-Wallis test and Dunn's post hoc test were performed for nonparametric data, according to the Kolmogorov-Smirnov normality test. Two-way ANOVA was performed to analyze differences in total area of fibrosis between the groups (green and red); *p* < 0.05 were considered significant in all cases. All statistical analyses were performed using GraphPad Prism 7.0 software.

## 3. Results

### 3.1. Liver Function Assessment

**Figure 2:**
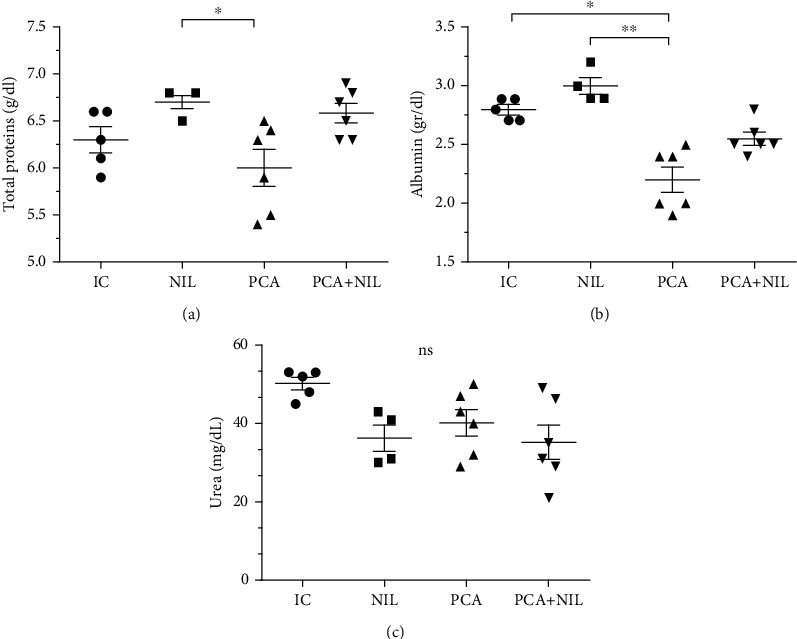
Effects of neurointermediate pituitary lobectomy (NIL), portacaval anastomosis (PCA), and PCA+NIL on total proteins (a), albumin (b), and urea (c) serum levels. The intact control (IC) group served as reference. (a) ^∗^*p* < 0.05: NIL vs. PCA+NIL. (b) ^∗^*p* < 0.05: IC vs. PCA and ^∗∗^*p* < 0.01NIL vs. PCA. (c) Nonstatistical (NS) differences between groups were observed. It was evaluated with analysis of variance test with the Tukey post hoc values, which are expressed as the mean ± SD.

As shown in Figure 2(a), serum levels of total proteins in the NIL group were not significantly different from the IC group. PCA induced a significant decrease in total proteins (*p* < 0.05) as compared with those in the NIL group, whereas the NIL surgery restored the serum proteins to normal in the PCA+NIL group (NS: NIL vs. NIL+PCA). The albumin serum levels were not significantly different in the IC, NIL, and PCA+NIL groups, while a significant decrease in the serum albumin occurred in the PCA group (*p* < 0.05 and *p* < 0.01: PCA vs. IC and NIL, respectively; Figure 2(b)). In the PCA+NIL group, the AVP deficiency caused a mild recovery in the albumin serum levels (NS: IC and NIL vs. PCA+NIL; Figure 2(b)). Compared with the IC group, NIL, PCA, and PCA+NIL did not affect the urea serum levels (Figure 2(c)).

**Figure 3:**
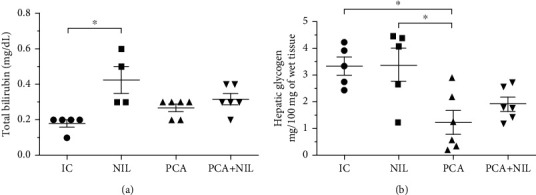
Effects of neurointermediate pituitary lobectomy (NIL), portacaval anastomosis (PCA), and PCA+NIL on (a) total bilirubin serum levels and (b) hepatic glycogen. ^∗^*p* < 0.05 IC vs. NIL. The intact control (IC) group served as reference. It was evaluated with analysis of variance test with the Tukey post hoc values, which are expressed as the mean ± SD.

In comparison with the IC group, no significant changes in total bilirubin serum levels occurred in the PCA and PCA+NIL groups. The NIL surgery alone caused a significant increase in the bilirubin serum levels in the NIL group (*p* < 0.05: IC vs. NIL) (Figure 3(a)). In comparison with the IC and NIL groups, the PCA surgery alone induced a significant decrease in the glycogen content (*p* < 0.05: IC and NIL vs. PCA group), whereas the NIL to the PCA+NIL group caused a mild recovery in the glycogen content (NS: IC, NIL, and PCA vs. PCA+NIL group) (Figure 3(b)).

**Figure 4:**
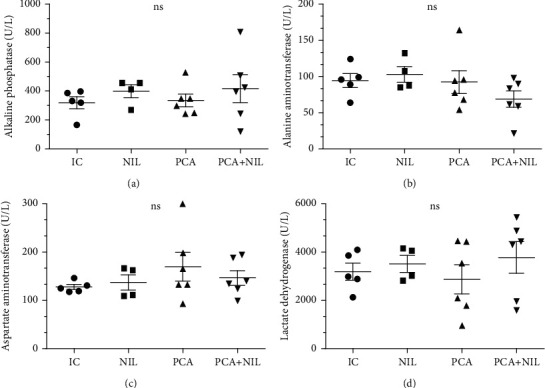
Effects of neurointermediate pituitary lobectomy (NIL), portacaval anastomosis (PCA), and PCA+NIL on (a) alkaline phosphatase (AP), (b) aspartate aminotransferase (AST), (c) alanine aminotransferase (ALT), and (d) lactate dehydrogenase (LDH) serum levels. Nonstatistical differences between groups were apparent. The intact control (IC) group served as reference. It was evaluated with analysis of variance test with the Tukey post hoc values, which are expressed as the mean ± SD.

The serum levels of the enzymes AP, AST, ALT, and LDH were not significantly affected by the different experimental conditions (Figures 4(a), 4(b), 4(c), and 4(d)). In summary, as shown in Figures 2, 3, and 4, four weeks of PCA is a model of liver damage, where the pathophysiological changes do not develop concurrently.

**Figure 5:**
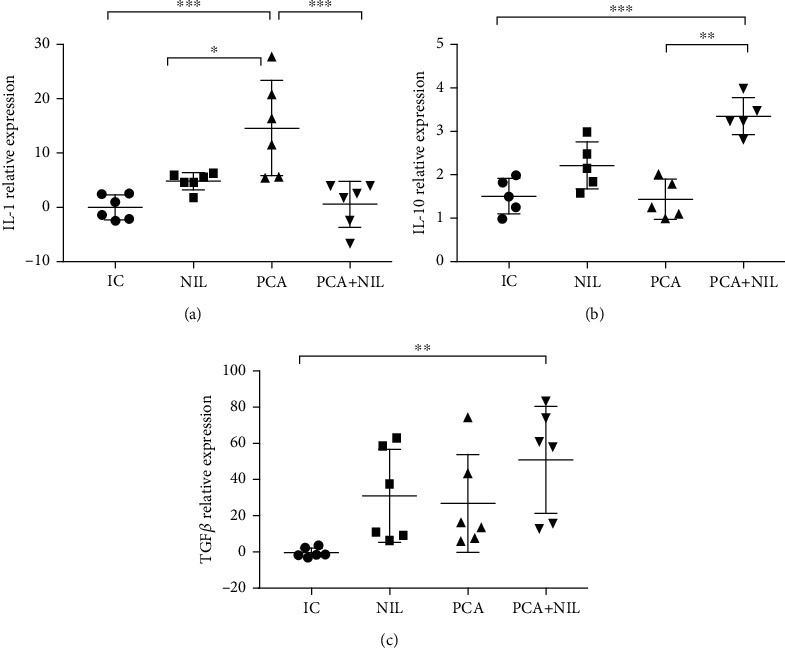
Effects of neurointermediate pituitary lobectomy (NIL), portacaval anastomosis (PCA), and PCA+NIL on the relative expression of (a) IL-1, (b) IL-10, and (c) TGF-*β*. (a) ^∗∗∗^*p* < 0.001: IC vs. PCA, ^∗^*p* < 0.05: NIL vs. PCA, and ^∗∗∗^*p* < 0.001: PCA vs. PCA+NIL. (b) ^∗∗∗^*p* < 0.001 IC vs. PCA+NIL. (c) ^∗∗^*p* < 0.01: IC vs. PCA+NIL. The intact control (IC) group served as reference. It was evaluated with analysis of variance test with the Kruskal-Wallis test and Dunn's post hoc values, which are expressed as the mean ± SD.

The relative gene expression levels of proinflammatory cytokine IL-1 and anti-inflammatory cytokines IL-10 and TGF-*β* showed that in comparison with the IC group, PCA induced a significant increase in IL-1 level (*p* < 0.001: IC vs. PCA), NIL surgery did not affect the IL-1 expression level, while the PCA+NIL group showed a diminution of the IL-1 expression levels to basal levels (Figure 5(a)). Compared with the IC, NIL, and PCA groups, the expression levels of the anti-inflammatory cytokines IL-10 and the TGF-*β* were significantly higher in the PCA+NIL group (*p* < 0.001 and *p* < 0.01; IC vs. PCA+NIL, respectively; Figures 5(b) and 5(c)).

**Figure 6:**
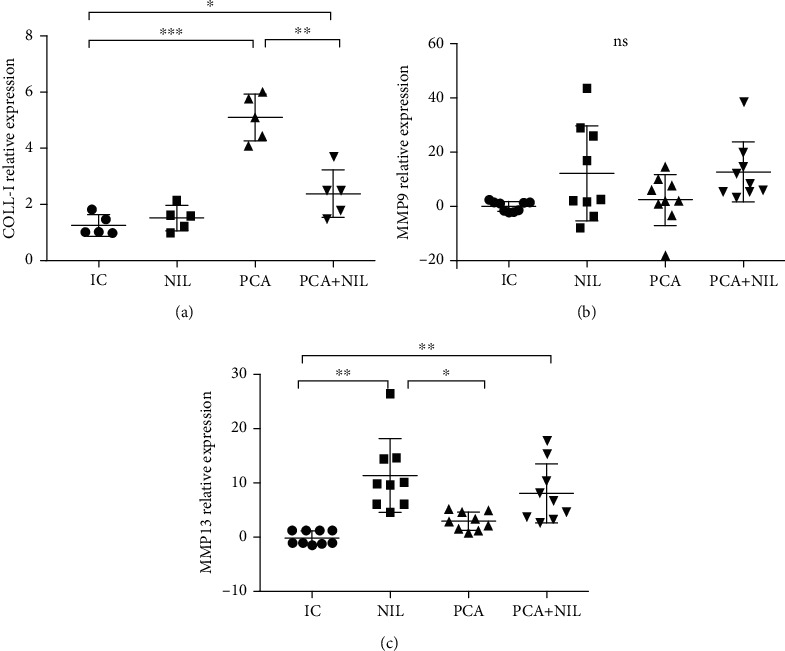
Effects of neurointermediate pituitary lobectomy (NIL), portacaval anastomosis (PCA), and PCA+NIL on the relative expression of (a) collagen type 1 (COLI), (b) metalloproteinase-9 (MMP-9), and (c) metalloproteinase-13 (MMP-13). (a) ^∗^*p* < 0.05: IC vs. PCA-NIL and ^∗∗^*p* < 0.01: IC vs. PCA. (b) Nonstatistical differences between groups were apparent. (c) ^∗∗^*p* < 0.01: IC vs. NIL, ^∗∗^*p* < 0.01: IC vs. PCA+NIL, and ^∗^*p* < 0.05: NIL vs. PCA. The intact control (IC) group served as reference. It was evaluated with analysis of variance test with the Kruskal-Wallis test and Dunn's post hoc values, which are expressed as the mean ± SD.

The relative gene expression levels of COLL-I, MMP-9, and MMP-13 showed that while PCA induced a significant increase of the COL-I expression level (*p* < 0.01: IC vs. PCA), the PCA+NIL group showed a decrease in COLL-I expression level, although not to the IC group levels (*p* < 0.05: IC vs. PCA+NIL group; Figures 6(a), 6(b), and 6(c)).

The MMP-9 was not significantly expressed in the NIL, PCA, and PCA+NIL groups compared with the IC group (Figure 6(b)). While the NIL and PCA+NIL groups showed significantly increased MMP-13 expression levels (*p* < 0.001: IC vs. NIL and PCA+NIL, respectively), no significant differences were found between the expression levels in the IC vs. PCA groups (Figure 6(c)).

### 3.2. Effects of NIL, PCA, and PCA+NIL on the Liver Histopathology

**Figure 7:**
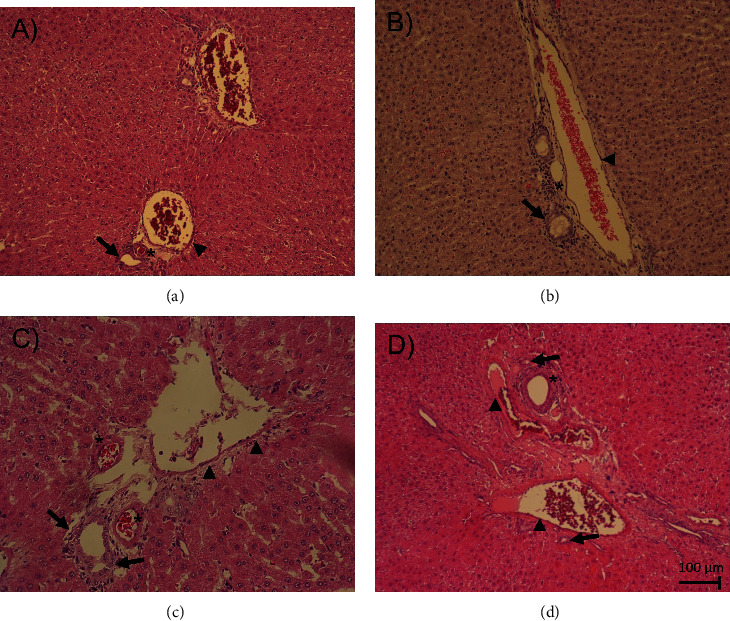
Liver slides HE stained from the IC, NIL, PCA, and PCA+NIL groups. (a) Slide from an IC animal. Normal pattern of the liver lobules, triads (hepatic artery ∗), bile ducts (arrow), portal vein (arrowhead), and blood sinusoids are apparent. Neither inflammatory infiltrates nor necrosis or fibrosis is present. (b) The NIL group. Similar normal structural patterns of the IC liver morphology are discernible. (c) PCA slide. Evident morphological changes are noted in the periportal zone, increased collagen deposits, thickening of the bile ducts (arrows), artery walls (∗), and portal veins (arrowhead). (d) PCA+NIL slide. A restored morphological pattern of hepatocytes and sinusoids (∗) is apparent, as well as slimming of the portal vein wall (arrowhead). All images taken at 20x magnification. *n* = 5 − 6 animals/group.

The livers from all the groups showed histopathological changes in response to different experimental conditions.  Table 2 summarizes the main stromal and cellular changes found in the different experimental groups.  Figure 7 shows liver slides stained with HE method at 20x magnification. As shown in Figure 7(a), a normal pattern of the blood sinusoids and tissue morphology from an IC group show normal liver lobules and triads: hepatic artery (asterisk), bile ducts (arrow), and portal vein (arrowhead). No inflammatory infiltrates, necrosis, or fibrosis was observed (Table 2). The NIL group (Figure 7(b)) showed a similar normal histological pattern as the IC group (Table 2). The PCA group showed significant changes in the morphology of liver structures, mainly in the periportal zone, with a significant thickening around the bile ducts (arrows), artery walls (asterisks), and portal veins (arrowhead) caused by increased collagen deposits (Figure 7(c)). Some inflammatory infiltrations were occasionally observed (Table 2). The PCA+NIL group showed a partial reversion in the stroma and cell patterns as compared to the PCA and IC groups (Figure 7(d)) (Table 2). The periportal area showed the restored morphological pattern of the hepatocytes and sinusoids and the slimming of the portal vein wall (arrowhead).

**Figure 8:**
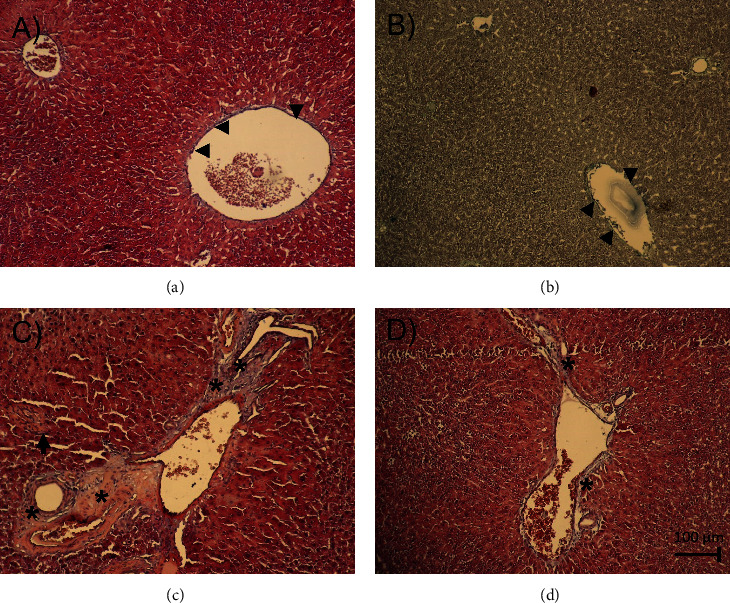
Liver slides stained with Masson's trichrome to identify collagen distribution (blue) in the several experimental groups. In (a) from an IC and (b) from a NIL group, a thin pattern of collagen fibers is distributed around the triad (hepatic artery, bile duct, and portal vein) (arrowhead). (c) PCA liver slide. An increase in collagen distribution around the vessels of the triad and a mild invasion into the liver parenchyma is observed (∗). In addition, isolated foci of inflammation are observed (arrow). (d) PCA+NIL liver slide. In comparison with the PCA group, the collagen distribution around the triad and liver parenchyma is less apparent (∗). All photographs are taken at 20x magnification.

Masson's trichrome staining method to assess the distribution of collagen fibers (blue) in the several liver groups showed major histopathological changes mainly in the periportal area of the PC and PC+NIL groups (Figure 8). The IC (Figure 8(a)) and NIL (Figure 8(b)) groups showed a thin pattern of collagen distribution around the portal vein (arrowhead). The effects of PCA were observed as an increased collagen deposition around the triad vessels and mildly into the surrounding liver parenchyma (asterisk in Figure 8(c)). In addition, isolated inflammatory infiltrates were observed (arrow). In comparison with the APC group (Figure 8(c)), the PCA+NIL group (Figure 8(d)) showed decreased collagen deposits around the triad and liver parenchyma (asterisk). All photographs were taken at 20x magnification.

**Figure 9:**
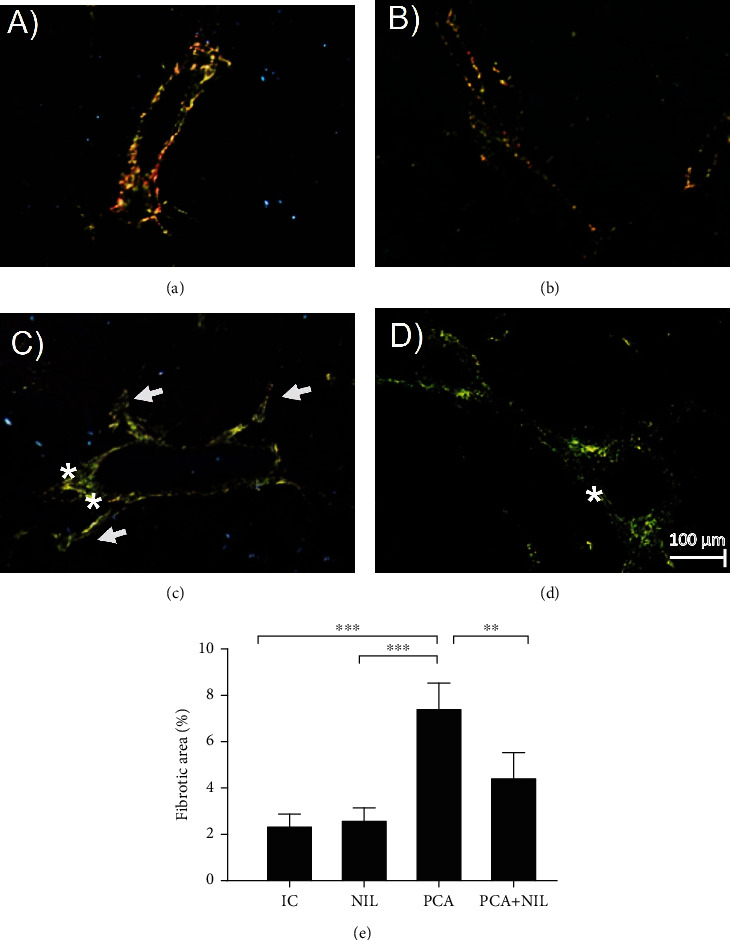
Liver slides stained with Sirius Red analyzed under polarized light to assess the area of fibrosis. (a) Periportal area from an IC animal. Normal distribution of type III collagen (mainly green color) is observed. (b) NIL liver slide. Similar distribution of collagens of the IC group is apparent. (c) PCA liver slide. Increase in collagen type I (red) and III (yellow) distribution is observed in the periportal area, with thickening of the triad vessels. Also, an increased collagen invasion into the surrounding liver parenchyma (∗) is apparent. (d) PCA+NIL. It shows regression of type I collagen, mainly in the periportal area (∗). (e) Comparisons of percentage of the fibrosis areas in the several experimental groups. Observe that between the IC and NIL groups, similar percentages of fibrosis are apparent. In contrast, the PCA animals developed a significant increase in the fibrotic area (∗∗*p* < 0.001: IC vs. PCA). In the PCA+NIL group, the percentage of fibrosis was significantly decreased as compared with the PCA group (^∗^*p* < 0.01: PCA vs. PCA+NIL group), indicating reversible effect of AVP deficiency on liver fibrosis. It was evaluated with analysis of variance test with the Tukey post hoc values, which are expressed as the mean ± SD. Pictures are taken at 20x magnification.

The slides stained with Sirius Red and analyzed under polarized light to assess the collagen types and areas of fibrosis showed normal basal distribution of type III collagen (mainly yellow and green colors) in the periportal area from an IC group (Figure 9(a)). The NIL group showed similar distribution of collagen, although thinner than that observed in the IC group (Figure 9(b)). In the PCA group, (Figure 9(c)) large changes in the distribution and type of collagens were observed such as increased fibrosis in the periportal area type III collagen (green, asterisk), thickness of the triad vessels, and increased collagen invasion into the surrounding liver parenchyma (arrow). PCA+NIL animal showed a decreased amount of collagen invading the liver parenchyma (type III collagen) and a significant diminution of the periportal fibrotic area (asterisk; Figure 9(d)). To assess the percentage of liver fibrosis, the ImageJ software program was used [36, 37]. On comparing the percentage area of fibrosis among the different groups, NIL surgery showed no effect on the percentage of fibrosis as compared to the IC group (Figure 9(e)). In contrast, the PCA group developed a significant increase in the fibrotic area (*p* < 0.001: IC vs. PCA, Figure 9(e)). The PCA+NIL group showed significantly lower percentage (*p* < 0.01: PCA vs. PCA+NIL group, Figure 9(e)).

Previously, it was demonstrated that NIL induced an immediate but transient increase in water intake and urine output (*diabetes insipidus*) for 2-4 weeks and a permanent drop in AVP serum levels. AVP assessed at 3, 15, 45, and 90 days after NIL were on average 2.4 ± 0.16 pg/mL versus 10.6 ± 0.08 pg/mL of their respective control groups [24]. Similar low AVP serum levels were also reported 3 and 8 weeks after NIL surgery [38].

## 4. Discussion

In the present work, PCA as a model of chronic liver disease is supported by the decreased circulating levels of total proteins and albumin, decreased liver glycogen level, the increased relative expression levels of IL-1 and COLL-I genes, nonsignificant changes in gene expression levels of MMP-13 and IL-10, and the significant increase in periportal (triads) and Rappaport parenchymal 1 and 2 fibrosis. This information, along with those previous findings of Aller et al. [13], Vázquez-Martínez et al. [14], and Gandhi et al. [39], reinforces that PCA is a paradigm of chronic liver damage.

The stimulating role of AVP in fibrotic process has been demonstrated in several clinical and experimental conditions. *In vitro*, AVP stimulates the mesangial cell proliferation, hypertrophy, type IV collagen production, and increased concentration of TGF-*β*, which are inhibited by the selective V1a AVP receptor antagonist (YM218) [30]. Experiments in rats and human observational studies suggest that AVP may play a role in the genesis and exacerbation of renal damage and chronic renal insufficiency [32]. Yan-Hong et al. [29] described the effect of AVP on cardiac fibroblast differentiation into collagen producer myofibroblasts, and Niu et al. [28] found a synergistic effect of angiotensin (fragments 1-7) and AVP on proliferation and collagen synthesis in rat cardiac fibroblasts. The presence of V1a and V2 AVP receptors in immune system cells and its stimulatory role during inflammatory responses has also been demonstrated [40]. AVP V1a receptors were present in blood monocytes, macrophages, splenic lymphocytes, and B cells; V2 AVP receptors in peripheral blood cell cultures; and V1b receptors in the thymus and spleen cells. Furthermore, the V1a AVP receptors in HSCs and their activation and differentiation into collagen producer myofibroblasts were demonstrated by Bataller et al. [31], whereas the presence of V1a and V2 AVP receptors and their activation by AVP in hepatocytes and cholangiocytes were described by Dünser and Westphal [41]. Together, this information strongly supports that AVP is directly involved in innate and acquired immunity, as well as in the activation and development of the fibrotic process. This view is supported by the present results in the AVP-deficient animals.

In the NIL group, AVP deficiency increased both total bilirubin serum levels and relative gene expression levels of the MMP-13, with no significant effects on the remaining biochemical and histopathological parameters. Although the mechanism by which AVP deficiency (NIL-induced) causes hyperbilirubinemia is not known, a possible explanation for this may be that AVP in the liver is involved in hepatocyte ureogenesis, glycogenolysis, neoglucogenesis, and cell regeneration through its V1a receptors [41], while the V2 AVP receptors regulate the biliary epithelium functions [42]. It is known that AVP stimulates efflux of the bile salts taurocholate and glycocholate in suspended hepatocytes, via its association with the AVP V1 receptors on hepatocyte membranes [43, 44]. It is also known that several hepatobiliary organic anion-transporting polypeptide systems (Oatps in rodents) located in the basolateral membrane extract chemicals from sinusoidal blood into the hepatocytes, while canalicular transporters mediate the movement of chemicals into the lumen of the bile canaliculus, including the bile acids and unconjugated bilirubin (in rodents) [45]. Therefore, we speculate that the hyperbilirubinemia in the NIL group may be due to the low AVP circulating levels, which were not enough to activate the AVP receptor signaling mechanisms that mediate the hepatocyte bilirubin excretion. Further experiments must be conducted to evaluate this possibility.

Results also show that one week of AVP deficiency in the PCA+NIL animals caused the following effects: (i) reversion of some of the altered metabolic parameters to normal (total proteins and serum albumin and liver glycogen content), (ii) increase in the anti-inflammatory IL-10 gene expression level, (iii) decrease of both COLL-I gene expression level and deposition of type I collagen, and (iv) increase in MMP-13 gene expression level and depressed liver fibrosis (assessed by histopathology). The anti-inflammatory and antifibrotic role of IL-10 has been associated with the control of inflammation in many organs with clinical diseases and experimentally induced fibrosis [46, 47]. Currently, the administration of IL-10 is considered as a tentative pharmacological tool in the treatment of liver inflammation and fibrosis [46, 48]. Considering all the previous observations, present results can be partially explained as follows: the low levels of AVP (NIL-induced) are not enough to activate the AVP receptors of the immune cells and HSCs, resulting in a decreased activation of the cell signaling pathways associated with the proinflammatory-profibrotic responses (IL-1, COLL-1). Simultaneously, the AVP deficiency activates cell signaling pathways associated with an anti-inflammatory-antifibrotic state (IL-10 and MMP-13), thus favoring a decreased liver inflammatory response and less activation of the HSCs and fibrosis, favoring liver recovery. This possibility is supported by our previous work on NIL-cirrhotic hamsters, in which both overexpression of MMP-13 and decreased expression level of TIMP-2 were accompanied by a significant regression in liver cirrhosis [33].

In response to acute and chronic liver injury, TGF-*β* is activated from the ECM deposits and then expressed and released from various cell types. The presence of V1a AVP and TGF-*β* receptors in the HSCs and its activation by inducing transdifferentiation of HSCs into collagen producer myofibroblasts have been demonstrated previously [31, 49]. In cooperation with other signaling pathways (reactive oxygen species (ROS), platelet-derived growth factor (PDGF), and connective tissue growth factor (CTGF)), the TGF-*β* signaling is considered the key fibrogenic factor in liver fibrosis [49]. In the present study, despite significant increase of TGF-*β* gene expression levels in the PCA+NIL animals, the inflammatory-fibrotic process (PCA-mediated) was downregulated. A possible explanation for this may be that the increased expression level of TGF-*β* combined with the inhibition in the expression of the IL-1 and COLL-1 (proinflammatory-profibrotic factors) was overcome by anti-inflammatory-antifibrotic factors (IL-10 and MMP-13). However, further experiments are required to establish this interpretation.

The effects of NIL on the adenohypophyseal hormone secretions have been reported [50–53]. Based on the literature and our previous work, the short- and long-term effects of the NIL surgery on several adenohypophyseal hormone secretions might be differentially regulated; thus, the main hormone secretory changes in response to NIL occur for a short time, returned to basal conditions after a few weeks, including the ability of the pituitary cells to respond to different physiological challenges [50–53]. In our studies, the effects of NIL on GH and TSH secretions decreased after 3 weeks of NIL and reverted to normal levels after eight weeks after surgery, whereas PRL, FSH, LH, and ACTH always showed normal ranges [27, 38, 53, 54]. Furthermore, to assess the viability of the physiological reactivity of the hypothalamic-pituitary-thyroid axis, NIL rats were simultaneously subjected to thyroidectomy. Results showed that NIL-thyroidectomized rats responded with a significant increase in TSH secretion levels, accompanied by significant changes in pituitary thyrotrophs, which underwent hypertrophy, hyperplasia, and development of thyroidectomy cells [53, 55]. These results suggest that short- and long-term endocrine effects of NIL on adenohypophyseal hormonal secretion are transient and that the regulatory hypothalamic-adenohypophyseal mechanisms for hormone secretions in the NIL animals were able to adapt to the permanent diminution of AVP and OT serum levels.

## 5. Conclusions

From the present study, we conclude that PCA is a good model to study chronic liver damage. The inflammatory and fibrotic effects of PCA are partially reverted by the AVP deficiency (NIL-induced) through both decreased expression of inflammatory-fibrotic factors and increased expression of the anti-inflammatory-antifibrotic factors, resulting in a decreased fibrosis and improvement in liver functions. Present results support the view that AVP plays a direct role in the regulation of the immune system and fibrotic process. Further experiments are required in order to obtain better insight regarding cell and molecular mechanisms through the AVP deficiency which stimulate or inhibit the cell signal pathways involved in the anti-inflammatory and antifibrotic processes responsible of improvement liver damage.

## Figures and Tables

**Table 1:** Oligonucleotide sequences.

Gene	Oligonucleotide-F	Oligonucleotide-R	Accession number
IL-1	5′-CTGTGACTCGTGGGATGATG-3′	5′-GGGATTTTGTCGTTGCTTGT-3′	NM_031512.2
IL-10	5′-GAATTCCCTGGGAGAGAAGC-3′	5′-CGGGTGGTTCAATTTTTCAT-3′	NM_012854.2
TGF-*β*	5′-GACTCTCCACCTGCAAGACCA-3′	5′-CGGGTGACTTCTTTGGCGTA-3′	AY550025.1
COL-1	5′-TTGACCCTAACCAAGGATGC-3′	5′-CACCCCTTCTGCGTTGTATT-3′	NM_053356.1
MMP-9	5′-CAGAAGCCCAAGGAAGAGTG-3′	5′-AGACCCACAGGAAACCACAG-3′	AJ438266.1
MMP-13	5′-ATCCCAGCTTAGGGCTCAAT-3′	5′-GGGAAAACAGCTACGCTGAG-3′	AY135636.1
*β*-Actin	5′-GTCGTACCACTGGCATTGTG-3′	5′-GCTGTGGTGGTGAAGCTGTA-3′	XM_032887061.1

**Table 2:** Table of parameters of magnitude of cell damage and stroma.

Experimental group	Stromal changes			Cell changes		
	Arterial wall thickening	Collagen fibers	Inflammatory infiltrate	Pleomorphism	Binucleation	Balonization
IC	-	-	-	-	-	-
PCA	++	+++	++	++	++	+++
NIL	-	-	-	-	-	-
PCA+NIL	++	+	+	++	+	++

-: parameter not found in histological preparations; +: magnitude of damage found in histological preparations.

## Data Availability

The data that support the findings of this study are available from the corresponding author, AQS, upon reasonable request.

## Abbreviations

PCA: Portacaval anastomosis
AVP: Arginine vasopressin
OXT: Oxytocin
NIL: Neurointermediate pituitary lobectomy
HSCs: Hepatic stellate cells
IL-I*β*: Interleukin-1*β*
IL-10: Interleukin-10
TGF-*β*: Transforming growth factor-*β*
MMP: Matrix metalloproteinase
AST: Aspartate aminotransferase
ALT: Alanine aminotransferase
TNF-*α*: Tumor necrosis factor-*α*
IL-8: Interleukin-8
PDGF: Platelet-derived growth factor
ET-1: Endothelin-1
*α*-SMA: Alpha-smooth muscle actin
CCl_4_: Carbon tetrachloride
AP: Alkaline phosphatase
LDH: Lactate dehydrogenase
Ig: Immunoglobulin
TIMPs: Tissue inhibitors of metalloproteinases
HE: Hematoxylin & eosin
COLL-I: Type I collagen
GH: Growth hormone
PRL: Prolactin
CRH: Corticotropin-releasing hormone
ACTH: Corticotropin
TSH: Thyrotropin
FSH: Follicle-stimulating hormone
LH: Luteotropic hormone.
